# A Sensitive Electrochemical Immunosensor Based on PAMAM Dendrimer-Encapsulated Au for Detection of Norfloxacin in Animal-Derived Foods

**DOI:** 10.3390/s18061946

**Published:** 2018-06-15

**Authors:** Bing Liu, Min Li, Yaoshuai Zhao, Mingfei Pan, Ying Gu, Wei Sheng, Guozhen Fang, Shuo Wang

**Affiliations:** State Key Laboratory of Food Nutrition and Safety, Ministry of Education of China, Tianjin University of Science and Technology, No. 29 The Thirteenth Road, Tianjin Economy and Technology Development Area, Tianjin 300457, China; lmlm0919@mail.tust.edu.cn (M.L.); 13132539770@mail.tust.edu.cn (Y.Z.); panmf2012@tust.edu.cn (M.P.); guying@mail.tust.edu.cn (Y.G.); shengwei@tust.edu.cn (W.S.); fangguozhen@tust.edu.cn (G.F.)

**Keywords:** electrochemical immunosensor, dendrimer, signal amplification, norfloxacin, animal-derived foods

## Abstract

In this work, a sensitive electrochemical immunosensor has been reported for the determination of norfloxacin in animal-derived foods. The poly (amidoamine) dendrimer encapsulated gold nanoparticles (PAMAM-Au) played dual roles in the proposed sensing platform which not only accelerated the electron transfer process of sensing, but also increased the efficiency of the immobilized antibody. The HRP-labeled antigen, as the signal labels in the immunosensor, was introduced to catalyze the following reaction of the substrate hydroquinone with the aid of H_2_O_2_ in the competitive reaction. On the basis of the signal amplification of PAMAM-Au, the signal intensity was linearly related to the concentration of norfloxacin in the range of 1 μg·L^−1^–10 mg·L^−1^. All the results showed that the proposed strategy with low LOD (0.3837 μg·L^−1^) and favorable recovery (91.6–106.1%) in the practical sample, and it could provide a suitable protocol for norfloxacin detection in animal-derived foods with high sensitivity, good accuracy, and stability.

## 1. Introduction

The development of quinolones, especially fluoroquinolone antibiotics, has a history extending back over 50 years [1]. Fluoroquinolones (FQs) are a group of synthetic antimicrobials to prevent and treat infections [2]. Norfloxacin (NOR,) as one of the fluoroquinolones (FQs), has been widely applied in medicine [3]. Their residual toxicity and resistance have a significant effect on food and the environment, which is paid widespread attention. According to EU regulations, the maximum limit allowed of quinolones in edible tissues (poultry and pork) is 50 μg·kg^−1^, and in dairy products is 75 μg·kg^−1^. Nowadays, there are several analytical methods for the detection of NOR, including spectrofluorometry [4], high-performance liquid chromatography-tandem mass spectrometry (HPLC-MS/MS) [5,6], UV–VIS spectrophotometry [7], and enzyme-linked immunosorbent assay (ELISA) [8]. However, the traditional methods have some limitations, such as expensive devices [9], they are time consuming, and have technical labor requirements. Therefore, it is critical to develop a simple, sensitive, and rapid method for the efficient determination of NOR [10].

Polyamidoamine (PAMAM) dendrimers, a new type of polymer with well-defined globular shape, multiple branched structure, and a large number of functional groups (amino groups or carboxyl groups) on its periphery has increasingly aroused concern in analytical fields [11,12]. In recent years, they have been widely used as templates to encapsulate nanoparticles, given the high loading capacity [13]. It has been reported that dendrimer-encapsulated Pt nanoclusters have been applied to the fabrication of non-enzymatic electrochemical sensors for the determination of cellular hydrogen peroxide [14]. Additionally, the large number of groups on its periphery enable PAMAM to anchor more molecules, such as antigens, antibodies, and aptamers. In the previous report, dendrimer-encapsulated Ru nanoparticles (Ru-PAMAM) were used to connect avidin to construct an ultrasensitive electrochemiluminescence immunosensor for the detection of 5-hydroxymethylcytosine [15]. Due to the good absorptive capacity and excellent electrical conductivity, gold nanoparticles have been widely applied to bioscience, clinical medicine, and medical testing [16,17]. The dendrimer-encapsulated gold nanoparticles (PAMAM-Au) were prepared by the reduction of chloroauric acid into the dendrimer interior [18]. The prepared PAMAM-Au does not only have a favorable loading capacity of other molecules but also increases the electrical signal, because of its conductive superiority of gold nanoparticles [19,20]. Immuno-competition mode, which is based on the specific competitive recognition effect of antibodies for antigens and labelled antigens or coating antigens is a typical valid detection method for small molecular substances. Immuno-competition mode provides a sensitive, specific, and robust means for the quantitative determination. 

In the study, we developed a sensitive electrochemical immunosensor based on PAMAM-Au nanocomposites for the determination of NOR in animal-derived foods. The *p*-aminobenzoic acid (*p*-ABA) was firstly attached to the surface of the electrode by electropolymerization. The as-prepared PAMAM-Au could be firmly immobilized by amine groups of PAMAM-Au and carboxyl groups of *p*-ABA. The PAMAM-Au could not only conjugate with antibodies easier, but also accelerate the electron transfer process of sensing. Subsequently, the analyte and HRP-labeled antigen were added together to compete with the capture sites of the antibody. HRP-labeled antigen, as the signal label in the immunosensor, was introduced to catalyze the reaction of hydroquinone and H_2_O_2_ in the competitive reaction. By the catalytic reaction of HRP in H_2_O_2_-HQ system, the electrode signals were amplified and measured. The developed electrochemical immunosensor can determine NOR with good sensitivity and accuracy.

## 2. Materials and Methods

### 2.1. Apparatus and Reagents

Cyclic voltammetry (CV), differential pulse voltammetry (DPV), and electrochemical impedance spectroscopy (EIS) were performed on a LK-2006 electrochemical workstation (Tianjin Lanlike Chemical and Electronic High Technology Co., Tianjin, China). A conventional three-electrode system containing a glassy carbon electrode (GCE, 4 mm diameter), a platinum electrode, and a saturated calomel electrode (SCE) as the working electrode, the auxiliary electrode, and the reference electrode, respectively. Transmission electron microscopy (TEM) (EM-2010FEF, JEOL, Tokyo, Japan) was used to observe the particle size of PAMAM-Au.

A fourth-generation dendrimer (4.0 G-PAMAM), chloroauric acid, 1-ethyl-3-(3-dimethylaminopropyl) carbodiimide (EDC), *N*-hydroxysuccinimide (NHS), bovine serum albumin (BSA), and horseradish peroxidase (HRP) were all purchased from Sigma Chemicals (St. Louis, MO, USA). *p*-Aminobenzoic acid (*p*-ABA) and hydroquinone (HQ) were purchased from Aladdin.(Shanghai, China) Potassium ferricyanide, potassium hexacyanoferrate, potassium chloride, disodium phosphate, sodium dihydrogen phosphate, sodium chloride were all obtained from Tianjin Chemical Reagent Factory (Tianjin, China). Doubly-distilled water (DDW) was obtained by a Millipore Milli-Q water system (Millipore, NY, USA).

The monoclonal antibody of NOR was prepared in our laboratory [21].

### 2.2. Preparation of HRP-NOR

A total of 7 mg of norfloxacin hapten was accurately weighed and dissolved in 300 μL of *N*,*N*-dimethylformamide (DMF) followed by the addition of 12.65 mg *N*-hydroxysuccinimide (NHS) and 22.70 mg *N*,*N*-dicyclohexylcarbodiimide (DCC). The mixture was then stirred at room temperature for 4–6 h. Subsequently, the reaction solution was centrifuged (5 °C, 10,000 rpm, 5 min) to remove the precipitate to obtain the product called the active ester. The solution of the active ester was slowly added into 3 mL of sodium bicarbonate solution containing 10 mg HRP under ice-cooling conditions. Then, the solution was dialyzed in phosphate buffer solution (pH 7.4, 0.01 mol·mL^−1^) at 4 °C. After dialysis, an equal volume of glycerol was added into the HRP-NOR solution and the obtained product was stored at −20 °C for further use.

### 2.3. Synthesis of PAMAM-Au Nanocomposites

The PAMAM-Au nanocomposites were prepared as follows: 2 mL of 0.07 mmol·L^−1^ PAMAM solution were mixed with 2 mL 1 mmol·L^−1^ HAuCl_4_ solution Then, 2 mL of 1 mmol·L^−1^ formic acid was added to the above mixed solution. Under the intensive reaction, Au were extracted into the interior of PAMAM, and the color of the solution was changed from yellow to red at once. The transmission electron microscopy (TEM) were employed to investigate the morphology and the particle size distribution of PAMAM-Au nanocomposites.

### 2.4. Fabrication of the Electrochemical Immunosensor 

**Scheme 1:**
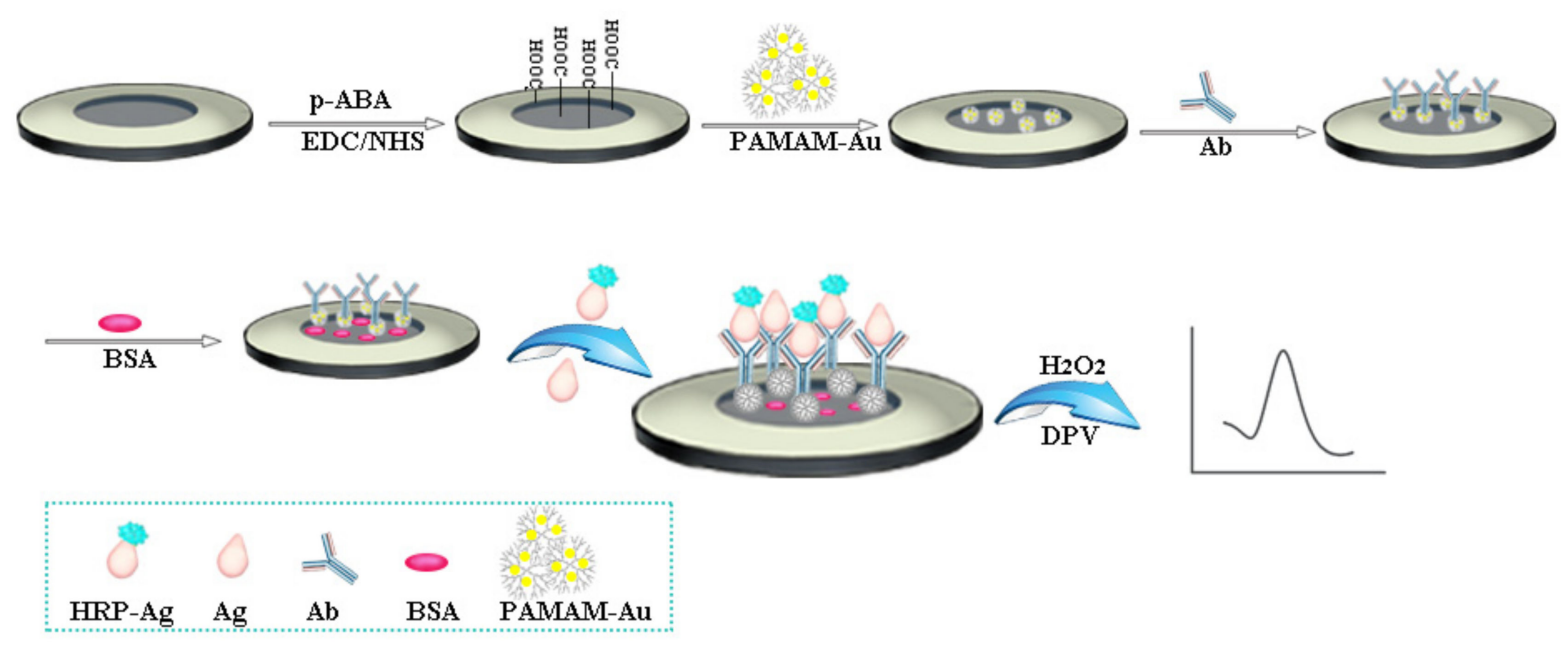
Schematic illustration of the preparation procedure of the electrochemical immunosensor for the detection of NOR.

The bare GCE was polished repeatedly with different amounts of alumina powder (1.0, 0.3, and 0.05 μm) and thoroughly cleaned by double-distilled water before use. The preparation of the immunosensor is illustrated in Scheme 1. Firstly, the cleaned GCE was scanned in 3 mL of 0.01 mol·L^−1^ HCl solution which containing 2 mmol·L^−1^ *p*-ABA by cyclic voltammograms in a potential range from 0 to 1.0 V with the scan rate of 40 mV·s^−1^. Secondly, a 10 μL EDC/NHS solution was casted on the surface of GCE for 30 min to activate the carboxyl groups and then the GCE was cleaned using the double-distilled water. After GCE was dried with nitrogen, 10 μL of PAMAM-Au were dropped onto the surface of *p*-ABA/GCE and dried naturally at room temperature. Finally, 10 μL 50 μg·mL^−1^ of NOR antibodies were added to the modified GCE and combined with PAMAM-Au thorough the formation of the amide bond. After that, 10 μL of 1% BSA solution was applied to deactivate the remaining amino groups and block the unreacted active sites. Ab/PAMAM-Au/*p*-ABA/GCE was then fabricated. The electrodes were washed with PBS after each modification step.

### 2.5. Measurement Procedure

Equal volume of enzyme-labeled antigen (HRP-Ag) and target with different concentration were mixed, respectively. Next, 10 μL of the mixtures were dropped onto the surface of the modified electrode which HRP-Ag and the target could competitively react with the antibodies. Finally, the prepared immunosensor was measured in a glass beaker with 3 mL of PBS (pH 7.4) solution which contained 3 mmol·L^−1^ hydroquinone (HQ) and 1.25 mmol·L^−1^ hydrogen peroxide (H_2_O_2_). The electrochemical response was recorded by differential pulse voltammetry (DPV). With the increasing concentration of the NOR, the current value gradually reduced and the quantitative detection of the NOR can be achieved.

### 2.6. Pretreatment of Real Samples 

In the experiment, three kinds of samples, including pork, egg, and milk, purchased from a local supermarket were chosen for spiked recovery experiments to evaluate the practicality of the established immunosensor for trace analysis of NOR. For the spiking study, the samples (5 g) were spiked with NOR dissolved in methanol to give concentrations of 50, 100, and 500 μg·kg^−1^. The extraction process was carried out using solid phase extraction (SPE) with a commercial C18 cartridge. Prior to use, the C18 cartridge was activated with methanol (6 mL) and distilled water (6 mL). The sample extract was loaded with a flow rate of 2.0 mL·min^−1^–3.0 mL·min^−1^, followed by washing with a solution of methanol/water (2 mL, 5%). The NOR remaining on the C18 cartridge was eluted with methanol (6 mL). The product was evaporated to dryness and reconstituted with methanol (1 mL) for binding measurements with the modified electrodes or HPLC-MS/MS analysis.

## 3. Results

### 3.1. Characterization of PAMAM-Au Nanocomposites 

**Figure 1:**
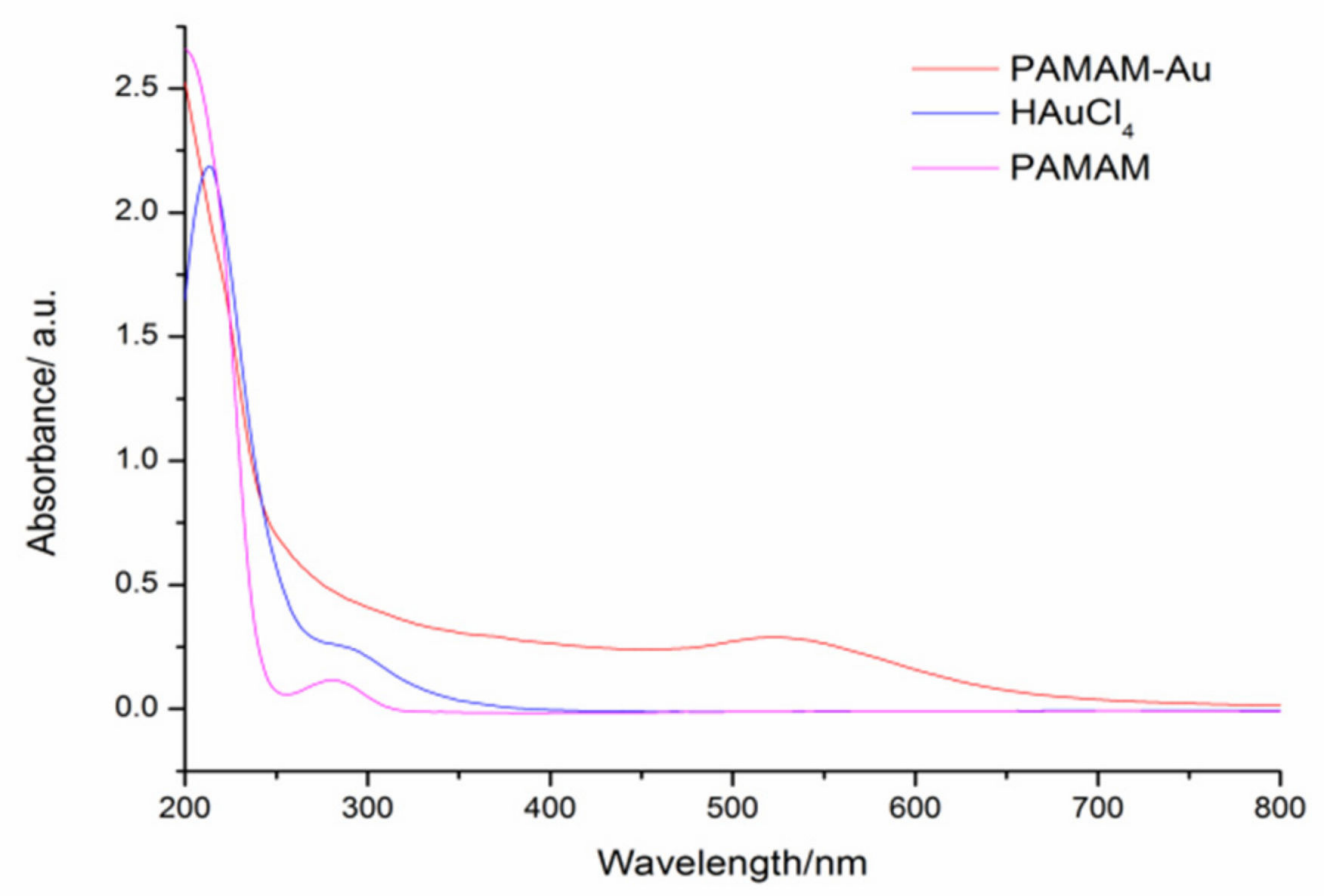
UV–VIS adsorption spectra of HAuCl_4_, PAMAM, and PAMAM-Au nanocomposites in their aqueous solution (1 mM HAuCl_4_, 0.07 mM PAMAM, and the 10-fold diluted as-synthesized PAMAM-Au).

**Figure 2:**
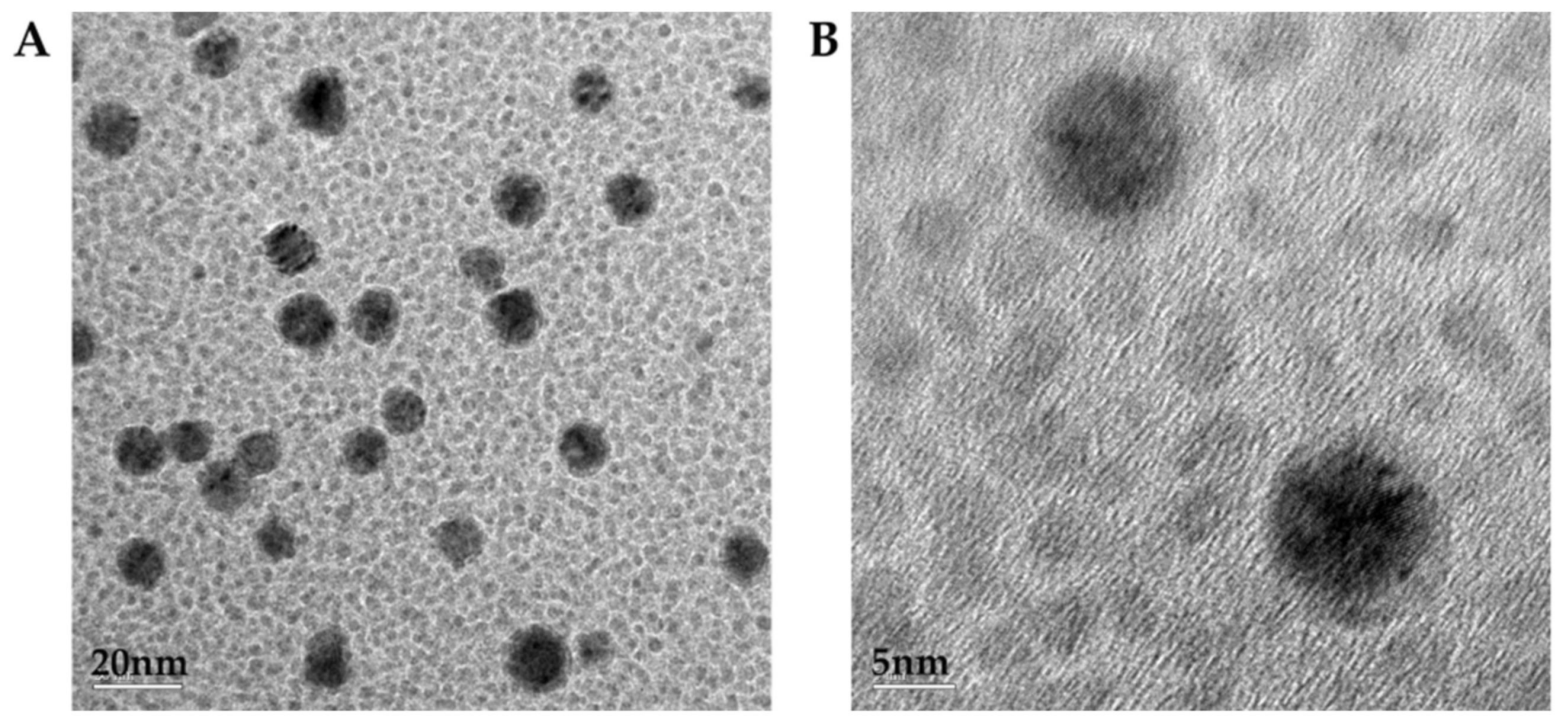
TEM image of PAMAM-Au at the scale of 1:20 (**A**) and 1:5 (**B**).

UV–VIS spectroscopy and transmission electron microscopy (TEM) were used to characterize the PAMAM-Au nanocomposites, respectively. From Figure 1, two strong absorption peaks of HAuCl_4_ are shown at 220 nm and 290 nm, and an absorption peak of PAMAM is shown at 290 nm [16]. While after reduction by the formic acid, a new absorption peak at 520 nm appeared indicating the generation of PAMAM-Au nanocomposites. From TEM in Figure 2, it was obviously found that PAMAM-Au nanocomposites (with the average 5 nm size) were dispersed uniformly.

### 3.2. Electropolymerization of p-ABA

**Figure 3:**
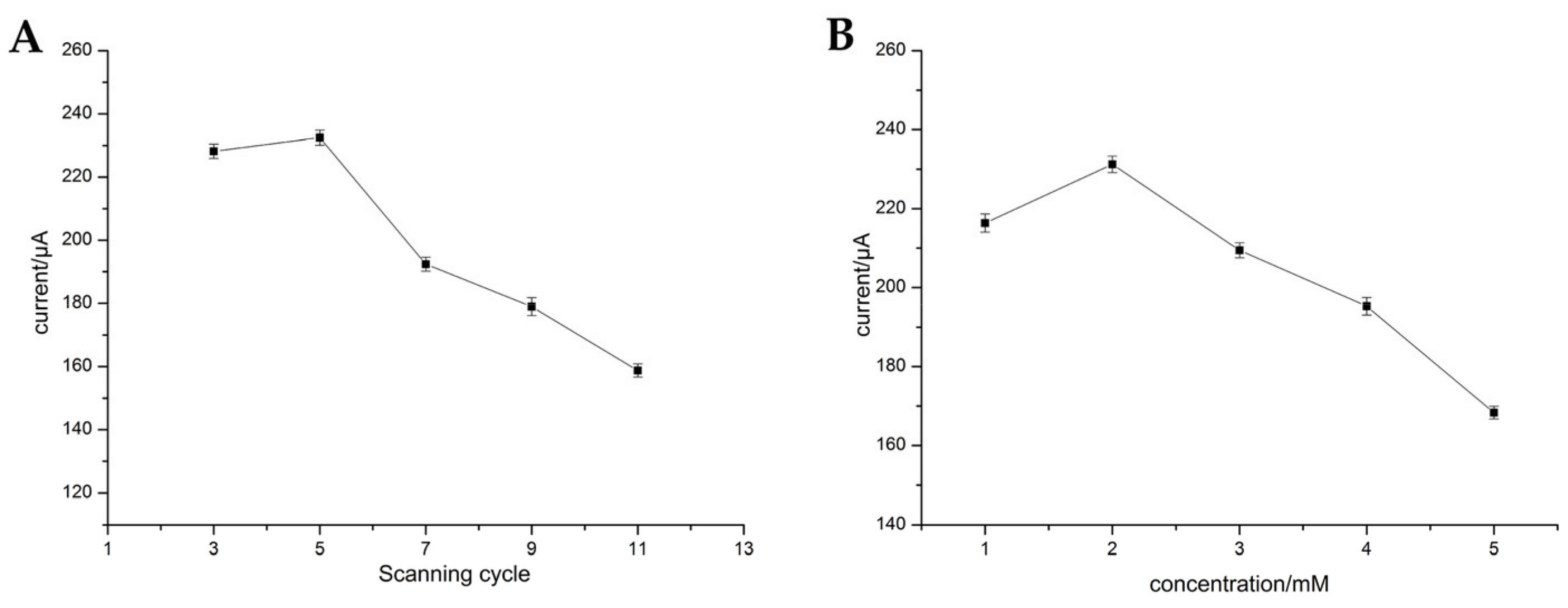
Dependence of the electrochemical signal on scanning cycle (**A**) and concentration (**B**) of *p*-ABA.

The *p*-ABA was firstly electropolymerized on the surface of the bare electrode to further covalently conjugate with PAMAM-Au. In order to obtain the optimal detection effect, the scanning cycles of electropolymerization and the concentration of *p*-ABA were studied. The different cycles of the polymerization were investigated from 3 to 11, and the results are shown in Figure 3A. We found that the current was increased as the cycles were added from 3 to 5, while when the cycles were over 5, the current was decreased. The specific reason is as follows: under the cycles of the polymerization from 3 to 5, the *p*-ABA fixed on to electrode surface was increasing, thus, the PAMAM-Au linked to *p*-ABA also was on the rise. With the further adding of electropolymerization cycles, PAMAM-Au linked to *p*-ABA reached the saturation point and excessive accumulation of *p*-ABA led to the excessive loading of carboxyl groups on the electrode surface, and the downward trend of the current attribute to the repulsive force between the [Fe(CN)_6_]^3−/4−^ probe and the negatively-charged carboxyl group of *p*-ABA. Hence, five cycles were the optimal condition of the electropolymerization process. Under the optimal cycle, the concentration of *p*-ABA was further studied in Figure 3B. The current was up to the maximum when the concentration of *p*-ABA was 2 mmol·L^−1^. Therefore, the optimal concentration of *p*-ABA was 2 mmol·L^−1^.

### 3.3. Optimization of the Immunoassay

**Figure 4:**
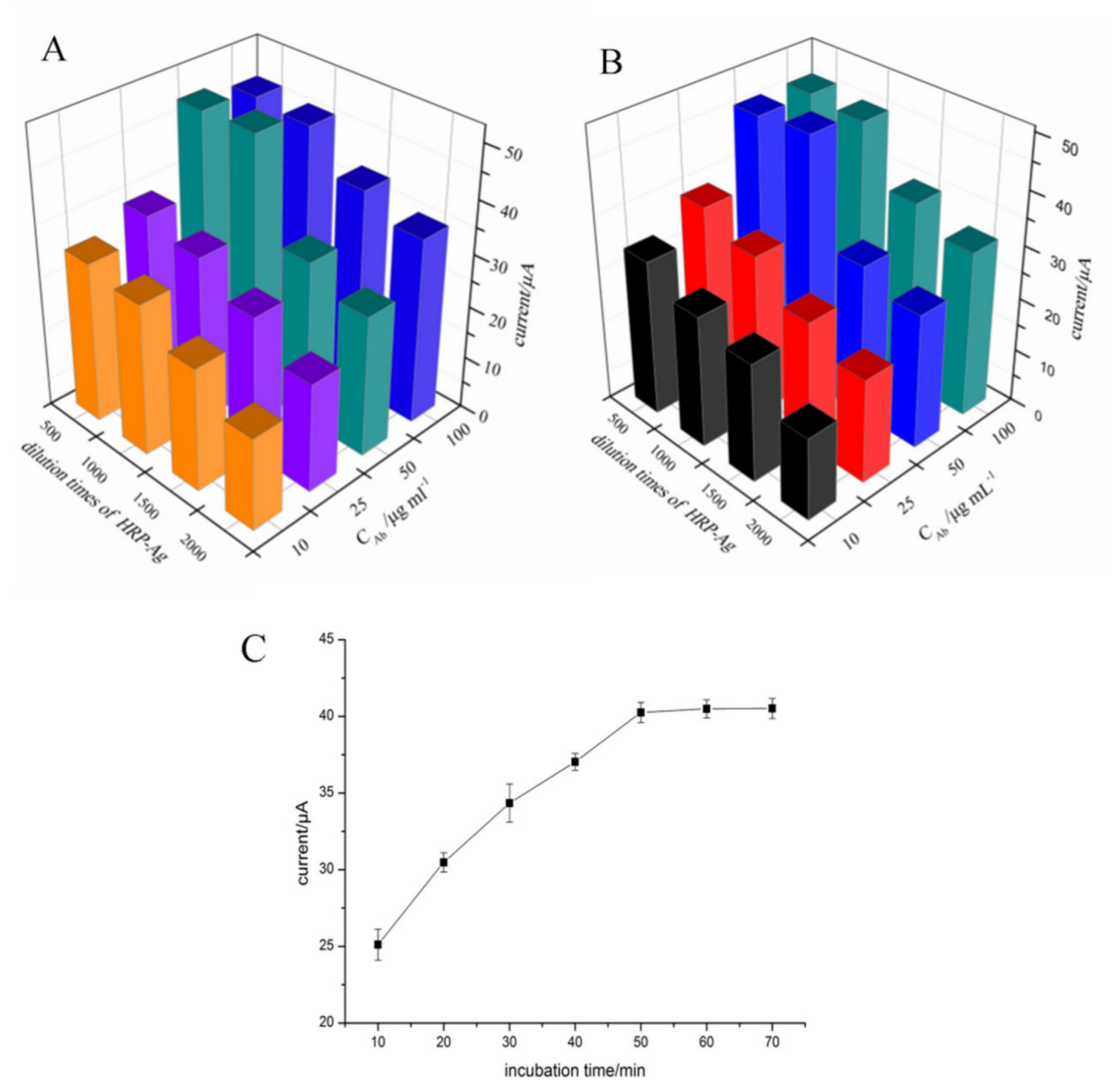
The response of Ab with different concentration and HRP-Ag with different dilution times in the absence of antigen (**A**) and in the presence of antigen (**B**); and dependence of the electrochemical signal on incubation time for the immunoreactions (**C**).

The added antigen and HRP-labeled antigen in the solution compete with the reaction sites on the antibody immobilized on the *p*-ABA modified electrode. Therefore, the concentration of antibodies and the dilution times of the HRP-labeled antigen were also optimized. In this study, we optimized the dilution times of HRP-Ag from 500 to 2000 times and the antibody concentration from 10 to 100 μg in the presence and in the absence of the antigen. The results are shown in Figure 4A,B, the maximum response was obtained under 50 μg·mL^−1^ of the antibody concentration, and 1000 times of the HRP-Ag dilution. The results show that the tendency of the DPV response in the presence and in the absence of the antigen are similar. In Figure 4B, the signal caused by the binding of the antibody and HRP-Ag in the presence of the antigen is lower than in the absence of the antigen (Figure 4A) due to the competition of antigen for the recognition sites on antibodies. Additionally, the optimal binding effect of antibodies and HRP-Ag were not affected by the adding of the antigen. Therefore, an Ab concentration of 50 μg·mL^−1^ and HRP-Ag dilution times of 1000 were used as the optimal conditions of the immune response. Furthermore, the incubation time for the immuno-competition interaction was also investigated. As can be seen in Figure 4C, with the increase of the incubation time from 10 to 50 min, the current response was increased and then tended to be stable when the time is over 50 min. Thus, 50 min was used as the optimal incubation time.

### 3.4. Optimization of Conditions for Electrochemical Detection

**Figure 5:**
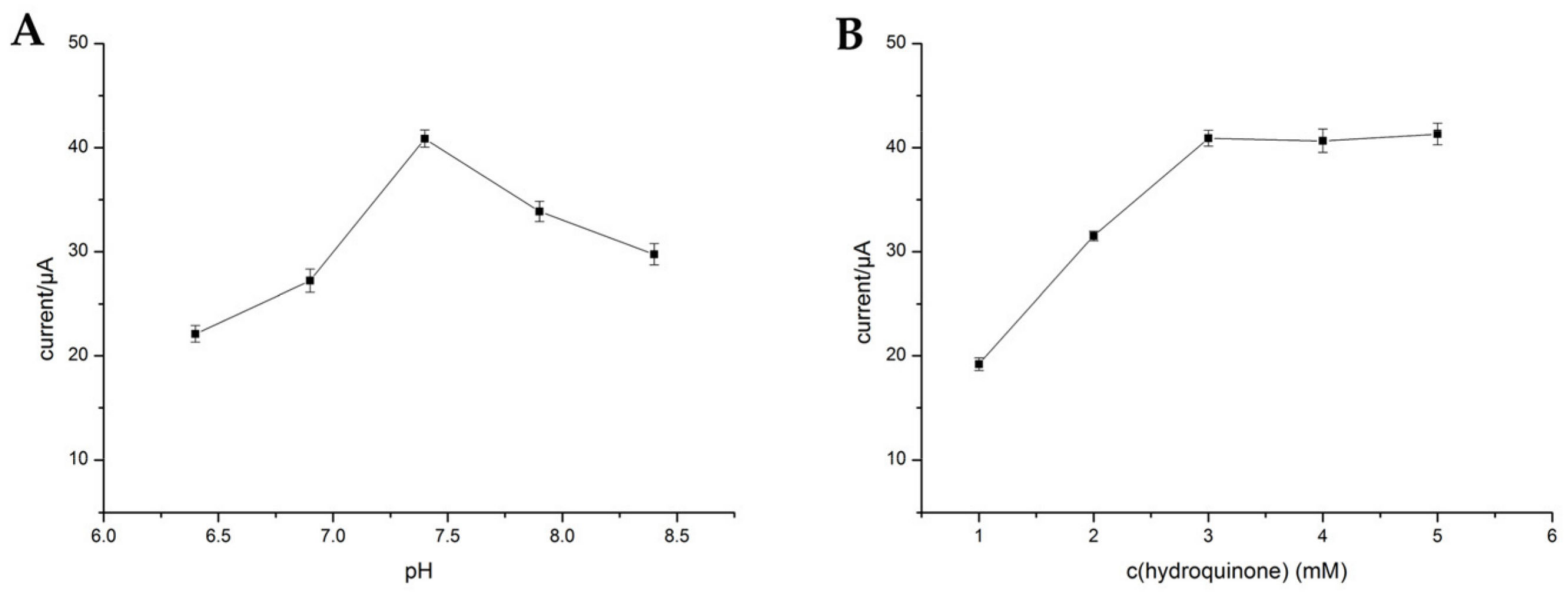
The influence of different pH (**A**) and the concentration of HQ (**B**).

The pH value and the concentration of HQ, had greatly affected the analytical performance in the process of the electrochemical detection. Here, the pH values (6.4, 6.9, 7.4, 7.9, 8.4, 8.9) were studied (Figure 5A). It was clearly observed that the current was increased when the pH increased from 6.4 to 7.4, and then decreased when the pH is over 7.4. Therefore, pH 7.4 which was close to the pH of human was selected. Figure 5B showed the effect of the different concentration of HQ for the catalytic reaction. The maximum current was achieved at 3 mmol·L^−1^, and then levelled off when the HQ concentration is over 3 mmol·L^−1^. Thus, 3 mmol·L^−1^ was used as the optical concentration of HQ.

### 3.5. Electrochemical Behavior of the Modified Electrodes

**Figure 6:**
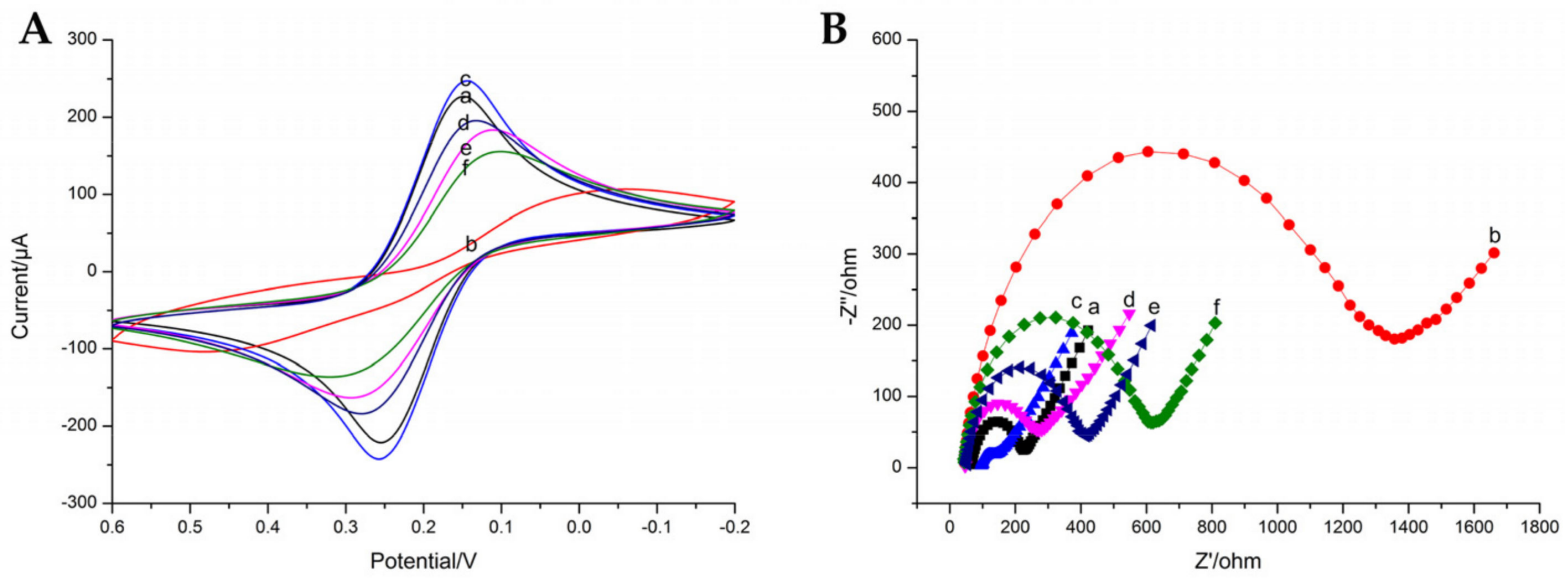
Cyclic voltammograms (**A**) and electrochemical impedance spectra (**B**) on (**a**) bare GCE; (**b**) *p*-ABA/GCE; (**c**) PAMAM-Au/*p*-ABA/GCE; (**d**) Ab/PAMAM-Au/*p*-ABA/GCE; (**e**) BSA/Ab/PAMAM-Au/*p*-ABA/GCE; and (**f**) Ag/BSA/Ab/PAMAM-Au/*p*-ABA/GCE in pH 7.4 PBS containing 0.2 M KNO_3_ and 5 mM Fe(CN)_6_^3−/4−^ as the redox probe.

CV and EIS can provide more detailed information on the preparation of the immunosensor. The CV and EIS behaviors of different modified electrodes were performed in 5 mM Fe(CN)_6_^3−/4−^ containing 0.2 M KNO_3_ (0.01 M pH 7.4 PBS). In Figure 6A, the CV profile clearly shows a pair of redox peaks of the bare GCE (curve a). After the electropolymerization of *p*-ABA, the current sharply dropped, demonstrating that *p*-ABA on the electrode can hinder electron transfer (curve b). When PAMAM-Au was covalently conjugated with *p*-ABA by the amido bond, the current value was obviously increased due to the favorable electrochemical performance of PAMAM-Au (curve c). After the antibody of NOR was immobilized on the electrode, the redox peak current was decreased due to the nonconducting property of antibodies, which hindered the electron transfer between the electrode and electrolyte solution (curve d). After blocking of the unbounded active sites by BSA, the redox peak current was further decreased (curve e), and the redox peak current was further decreased after the competition reaction, indicating that the antibody was specifically recognized for the antigen and HRP-Ag (curve f).

Electrochemical impedance spectroscopy (EIS) is an excellent and ultrasensitive technique to monitor the impedance variation of the biosensor during the modification procedures. The impedance spectra of step-by-step fabrication are represented in Figure 6B, which show the same tendency as the CV curves. The electron-transfer resistance (*R_et_*) for bare GCE is about 200 Ω (curve a). After the modification of *p*-ABA, the *R_et_* was increased to 1400 Ω (curve b). Then, the *R_et_* decreased obviously (curve c) due to the modification of PAMAM-Au on the electrode, and the *R_et_* was apparently increased when the antibody was immobilized on the electrode (curve d), which suggested that the modification of antibody on the electrode hindered the electron transfer of the Fe(CN)_6_^3−/4−^. After BSA was assembled on the electrode, the *R_et_* was further increased (curve e). Further incubation with antigen and HRP-Ag caused a further increment of *R_et_* (curve f).

### 3.6. Electrochemical Measure

**Figure 7:**
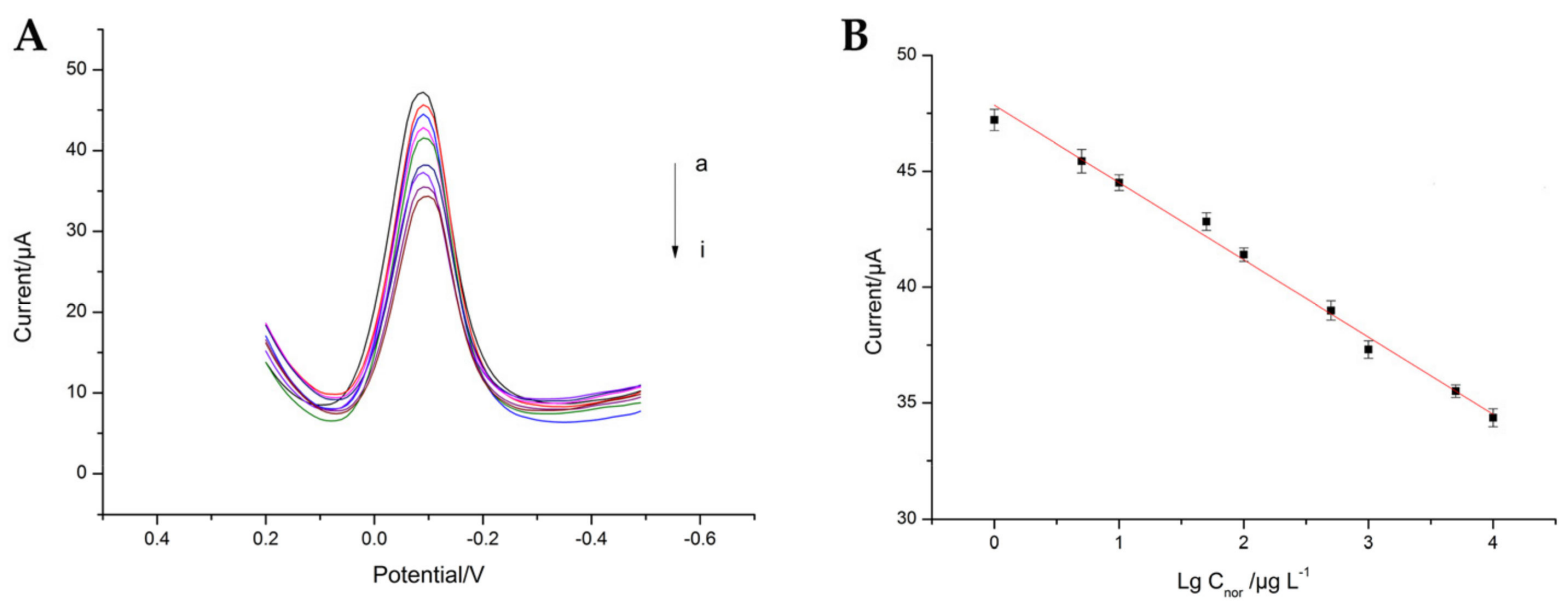
DPV peak current (**A**) and calibration curve (**B**) of the immunosensor at different concentrations of NOR.

Under the optimal conditions, the performance of the electrochemical immunosensor was investigated in a series of NOR standard solutions. Figure 7A shows the typical DPV peak current of immunosensor in the presence of different concentrations of NOR. As we can see, the peak current gradually decreased with the increasing concentration of NOR (curve a–i). Figure 7B shows a linear relationship between the DPV current and the logarithmic value of NOR concentration at the range from 1 μg·L^−1^ to 10 mg·L^−1^. The linear regression equation was I = −3.3344 lg Cnor + 47.84746 with a correlation factor of 0.9924, where the unit of I is μA and the unit of C is ng·L^−1^. The detection limit of the system was 0.3837 μg·L^−1^ (S/N = 3).

Furthermore, compared with other detection measures (Table 1), the fabricated immunosensor in this study possessed a low detection limit, wide linear range, and good recovery, further indicating that the immunosensor presented excellent properties. 

### 3.7. Evaluation of the Immunosensor 

**Figure 8:**
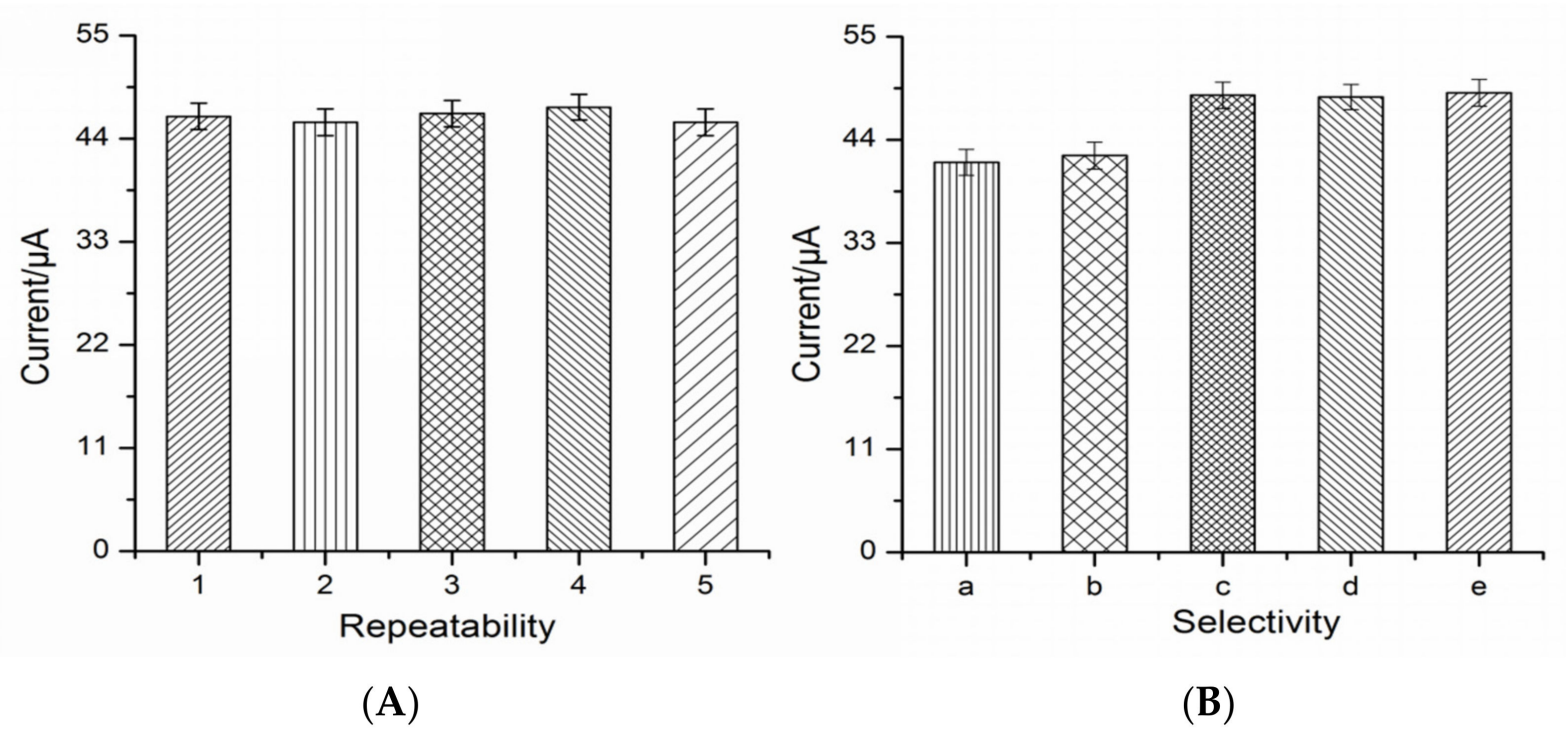
The repeatability of five modified electrodes (**A**) and the selectivity of the immunosensor with norfloxacin (**a**) and some analogues involving enoxacin (**b**), fleroxacin (**c**), mariposide (**d**), and sparfloxacin (**e**) (**B**).

The reproducibility of the immunosensor was investigated by determining NOR at a fixed concentration (10 μg·L^−1^) with five immunosensors which were fabricated independently. Figure 8A shows the acceptable relative standard deviation (RSD = 1.46%) indicating that the prepared electrochemical immunosensor had good reproducibility for NOR sensing. 

To test the selectivity of the immunosensor, the cross-reaction experiments were performed by other fluorequinolones involving enoxacin, fleroxacin, mariposide, and sparfloxacin (Figure 8B). The current response of enoxacin was close to that of NOR, but there was a gap between the other three analogues. It can be speculated that the cross-reaction between NOR and enoxacin may be attributed to their similar chemical structure. 

In stability studies, the modified electrode is stored at 4 °C for 3, 6, 9, 15, and 21 days, respectively. The response value of immunosensor after storage were obtained by 98.2%, 96.4%, 93.5%, 90.2%, and 88.1% in comparison with that of the first day, suggesting the good stability of the prepared electrochemical immunosensor.

### 3.8. Application of the Immunosensor to Daily Animal-Derived Food

The developed immunosensor was used to detect the NOR level in three daily animal-derived foods (milk, egg, pork). The samples were spiked with three different concentrations of NOR (50, 100, 500 μg·kg^−1^), then were determined by the prepared immunosensor and LC-MS standard method, respectively. The analytical results are listed in Table 2. As we can see, the recoveries that were obtained by the prepared immunosensor were within 91.6–106.1% with RSD below 4.2%. The result shows that the value obtained from the proposed method shows good consistency with that of LC-MS.

## 4. Conclusions

In this study, an electrochemical immunosensor based on immuno-competition mode was constructed for NOR detection and successfully applied in animal-derived foods samples. In the testing process, PAMAM-Au exerted a crucial function on the development of the effective biosensing platform, which not only enhanced the electrochemical sensitivity, but also could provide an adequate loading site for antibodies and further attributed to specific recognition of NOR. The HRP-labeled antigen was involved as a signal-trigger in H_2_O_2_ and the HQ catalytic system for quantitative analysis of NOR. All the results showed that the proposed approach with the preponderance of wide detection range (1 μg·L^−1^ to 10 mg·L^−1^) and low LOD (0.3837 μg·L^−1^) and favorable recovery (91.6–106.1%) in the practical sample could accurately and efficiently be applied to NOR monitoring. The proposed immunosensor showed a satisfactory stability for several repeated detectiosn and long-term storage demonstrating its promising application for NOR detection in daily animal-derived food.

## Figures and Tables

**Table 1:** Comparison of this method with other methods reported in the literature.

Methods	Linear Range (μg·L^−1^)	LOD (μg·L^−1^)	Recovery (%)	Reference
ELC	16–31,900	1.53	92.7–97	Deng et al. [22]
HPLC	-	1	95.2–97.6	Lagana et al. [23]
icELISA	-	0.05	77.3–117.8	Peng et al. [8]
CdTe QPs	100–90,000	250	93.0–104	Hong et al. [24]
Immunoassay	0.07–31.8	0.04	-	Dumont et al. [25]
Immunosensor	1–10,000	0.3837	91.6–106.1	This work

**Table 2:** Comparing of the results with the proposed immunosensor and the HPLC-MS/MS method in the detection of norfloxacin in daily animal-derived food.

Sample	Initial Concentration (μg·kg^−1^)	Added Concentration (μg·kg^−1^)	The Proposed Immunosensor		HPLC-MS/MS	
			Found (μg·kg^−1^)	Recovery ^a^ (%) (means ± SD, n = 3)	Found (μg·kg^−1^)	Recovery ^a^ (%) (means ± SD, n = 3)
Milk	10.5	50.0	59.70	98.4 ± 1.3	58.63	96.3 ± 1.2
		100.0	105.42	94.9 ± 2.6	109.88	99.4 ± 2.6
		500.0	539.52	105.8 ± 4.2	544.94	106.9 ± 0.8
Egg	6.8	50.0	53.17	92.7 ± 4.2	55.57	97.5 ± 1.7
		100.0	101.38	95.6 ± 1.2	105.51	98.7 ± 1.3
		500.0	537.25	106.1 ± 2.6	539.85	106.6 ± 1.6
Pork	7.4	50.0	53.21	91.6 ± 2.9	56.39	98.0 ± 4.4
		100.0	102.66	95.3 ± 1.2	104.36	96.9 ± 1.2
		500.0	521.85	102.9 ± 3.4	525.17	103.6 ± 1.8

^a^ Recovery is pointed to the additive recovery.
